# Pig regulatory macrophages as a donor-derived immune modulators in xenotransplantation

**DOI:** 10.3389/fimmu.2025.1718937

**Published:** 2025-12-05

**Authors:** Phu Chi Vu, Nhat Minh Dang, Vinh Phuoc Nguyen, Jonghyeok Jung, Joohyun Shim, Jeong Ho Hwang, Thi Xoan Hoang, Ik Jin Yun, Jae Young Kim

**Affiliations:** 1 Department of Life Science, Gachon University, Seongnam, Kyeonggi-Do, Republic of Korea; 2 Department of Transgenic Animal Research, Optipharm Inc., Cheongju, Republic of Korea; 3 Center for Bio-Signal Research, Division of Advanced Predictive Research, Korea Institute of Toxicology (KIT), Daejeon, Republic of Korea; 4 Nguyen Tat Thanh (NTT) Hi-tech Institute, Nguyen Tat Thanh University, Ho Chi Minh City, Vietnam; 5 Department of Surgery, Konkuk University School of Medicine, Seoul, Republic of Korea

**Keywords:** pig regulatory macrophages, xenotransplantation, inflammation, immunomodulation, coagulation

## Abstract

**Introduction:**

Xenotransplantation offers a potential solution to the critical shortage of donor organs; however, graft survival is limited by xeno-immune activation and coagulation dysregulation. Regulatory macrophages (Mregs) are known for their immunomodulatory capacity, yet their cross-species function in the context of xenotransplantation remains unclear. This study investigates the immunoregulatory properties of pig-derived Mregs (pMregs) as a potential donor-derived cellular therapy.

**Methods:**

pMregs were generated from pig CD14⁺ monocytes using M-CSF and IFN-γ. Phenotypic characterization was performed by flow cytometry, and functional assays evaluated cytokine secretion, T-cell suppression, and induction of FOXP3⁺ regulatory T cells across pig, monkey, and human lymphocytes. An *in vitro* xenogeneic inflammation model was established by exposing human M1 macrophages to pig endothelial cells to assess inflammatory cytokine responses and expression of coagulation-related genes.

**Results:**

pMregs exhibited a canonical Mreg phenotype (CD14⁺CD16⁺CD163⁺PD-L1⁺DHRS9⁺CD32⁻CD169⁻) and secreted IL-10 and TGF-β. Functionally, pMregs suppressed T-cell proliferation across pig, monkey, and human species and induced FOXP3⁺ regulatory T cells. In the xenogeneic inflammation model, pMregs attenuated inflammatory cytokine production in human M1 macrophages and downregulated mRNA expression of coagulation-associated genes, including TF and PAR-1.

**Discussion:**

These findings highlight the cross-species immunosuppressive activity and coagulation-regulatory capacity, suggesting the potential relevance to xenograft injury. pMregs may therefore serve as a promising candidate for further investigations as a donor-derived immunoregulatory cell type to complement genetically engineered pig grafts.

## 1 Introduction

Xenotransplantation, the transplantation of cells, tissues, or organs across species, offers a promising strategy to address the persistent shortage of donor organs. Among potential donors, pigs are considered the most suitable because of their large litter size, rapid growth, anatomical and physiological similarity to humans, and amenability to precise genetic modification (1). Advances in CRISPR-Cas technology have enabled the generation of pigs with reduced immunogenicity, bringing the field of xenotransplantation closer to clinical application (2–4).

Despite these advances, xenotransplantation still faces significant immunological barriers, including hyperacute rejection (HAR), acute vascular rejection (AVR), and acute cellular rejection (ACR) (1). These processes are driven by innate and adaptive immune responses involving preformed antibodies, complement activation, T cells, and macrophages (5–8). In addition, incompatibilities between pig and primate coagulation pathways contribute to ongoing thrombotic complications, which are further exacerbated by chronic systemic inflammation and elevated cytokines such as IL-6 (9, 10). Although genetic engineering of donor pigs has reduced antibody-mediated rejection, chronic inflammation and cell-mediated responses remain formidable obstacles (11–14).

Cell-based immunoregulatory approaches represent an appealing alternative to conventional immunosuppression. Among these, regulatory macrophages (Mregs) are a distinct subset with both antigen-presenting and immunosuppressive properties. Human Mregs, generated from CD14^+^ monocytes by macrophage colony-stimulating factor (M-CSF) and by interferon-gamma (IFN-γ), have shown potential in promoting graft tolerance in allotransplantation (15, 16). In kidney, heart, skin, and composite tissue transplant models, Mregs have demonstrated the ability to extend graft survival and reduce the requirement for long-term immunosuppression (15, 17, 18).

Although most studies of Mregs have focused on suppressing T-cell proliferation within the allogeneic setting, several lines of evidence suggest that donor-derived myeloid cells can exert regulatory effects across species. Notably, a landmark xenotransplantation study demonstrated that transplantation of donor pig thymus into baboons induced mixed chimerism and markedly prolonged pig kidney xenograft survival (19). This finding indicates that pig antigen-presenting and tolerogenic stromal cells are capable of interacting with primate immune cells in a regulatory manner, supporting the broader concept that pig immune-regulatory cells can shape primate immune responses. In addition, a tight crosstalk between inflammation and coagulation is a well-recognized driver of xenograft injury. Human Mregs have been reported to downregulate tissue factor and other thrombo-inflammatory mediators in activated macrophages, suggesting that modulation of the inflammation-coagulation axis is a general feature of Mregs (20). Given the evolutionary conservation of these pathways and the central role of macrophage-driven thromboinflammation in xenotransplantation, it is plausible that pig Mregs may exert similar regulatory effects on downstream pro-coagulant responses.

Therefore, we hypothesized that donor-derived pig Mregs (pMregs) could represent a novel solution to xenotransplantation-specific barriers by simultaneously suppressing xeno-immune activation and downregulating pro-coagulant responses. To test this, we generated pMregs from pig peripheral blood monocytes and evaluated their phenotypic, functional, and cross-species immunoregulatory properties in the context of xenogeneic cellular interactions.

## 2 Materials and methods

### 2.1 Cell culture

The human acute monocytic leukemia cell line THP-1 (Korean Cell Line Bank, Seoul, Korea) was maintained in RPMI-1640 medium (Welgene) supplemented with 10% heat-inactivated fetal bovine serum (FBS; Gibco), 100 U/mL penicillin (Gibco), and 100 μg/mL streptomycin (Gibco) prior to differentiation into macrophages.

The established pig vascular endothelial cell line MPN-3 (21) was cultured in DMEM (Welgene) containing 10% heat-inactivated FBS, 100 U/mL penicillin, and 100 μg/mL streptomycin. Human umbilical vein endothelial cells (HUVECs) were maintained in endothelial growth medium (EGM-2; Lonza, Walkersville, MD, USA) supplemented according to the manufacturer’s instructions. Jurkat T cells (E6.1; ATCC) were cultured in RPMI-1640 medium with 10% heat-inactivated FBS, 100 U/mL penicillin, and 100 μg/mL streptomycin prior to co-culture with pig regulatory macrophages.

### 2.2 Generation of monocyte-derived cells

pMregs and pig classically activated macrophages (pM1) were generated from pig CD14^+^ monocytes. CD14^+^ monocytes were isolated from PBMCs by positive selection with anti-CD14 microbeads (Miltenyi) and plated in six-well Cell+ culture plates (SPL Life Sciences, Korea) at 1 × 10^6^ cells per well in RPMI-1640 supplemented with 10% heat-inactivated pig serum (Gibco), 2 mM GlutaMAX (Invitrogen), 100 U/mL penicillin, 100 μg/mL streptomycin, and 5 ng/mL recombinant human M-CSF (rhM-CSF; R&D Systems) at 37°C, 5% CO_2_. After 4 days, cells were stimulated with 25 ng/mL recombinant pig IFN-γ (rpIFN-γ; R&D Systems) for 18–24 h to induce the pMreg phenotype.

For pM1 generation, CD14^+^ monocytes were cultured in RPMI-1640 containing 10% heat-inactivated pig serum, 2 mM GlutaMAX, antibiotics, and 50 ng/mL rhM-CSF. After 4 days, cells were further stimulated with 100 ng/mL rpIFN-γ and 100 ng/mL LPS (Sigma-Aldrich) for 18–24 h. THP-1 cells were maintained in RPMI-1640 containing 10% heat-inactivated FBS, 100 U/mL penicillin, and 100 μg/mL streptomycin. For differentiation into macrophages (hM0), cells were treated with 10 ng/mL phorbol 12-myristate 13-acetate (PMA, Sigma-Aldrich) for 2 days at 37°C in 5% CO_2_. To induce a classically activated phenotype (hM1), cells were additionally treated with 100 ng/mL LPS and 20 ng/mL recombinant human IFN-γ (rhIFN-γ; R&D Systems).

### 2.3 Isolation of pig CD14^+^ monocytes and pig and monkey CD4^+^ T cells

Blood collection was performed by jugular venipuncture using sterile, endotoxin-free techniques. All animals were handled in accordance with institutional and national guidelines for the care and use of laboratory animals.

Pig peripheral blood mononuclear cells (PBMCs) were isolated from the peripheral blood of healthy wild-type pigs (*Sus scrofa domesticus*, n = 3) obtained from Optipharm (Korea). All animal procedures were approved by the Institutional Animal Care and Use Committee (IACUC) of Optipharm under approval number OPTI-IAC-2405.

Pig CD14^+^ monocytes were purified from PBMCs by positive selection using anti-CD14 microbeads (Miltenyi Biotec, Bergisch Gladbach, Germany) according to the manufacturer’s instructions. The CD14-negative fraction was subsequently used for the isolation of pig CD4^+^ T cells. For this, cells were incubated with a biotin-conjugated anti-pig CD4 antibody followed by anti-biotin microbeads (Miltenyi Biotec) and positively selected using magnetic separation. The purity of the isolated cell populations was assessed by flow cytometry.

Monkey CD4^+^ T cells were isolated from PBMCs of *Cynomolgus macaques* (n = 2) by magnetic separation using human CD4 MicroBeads (Miltenyi Biotec) according to the manufacturer’s instructions. Briefly, PBMCs were incubated with CD4 MicroBeads for 15 min at 4°C, washed with MACS buffer (PBS supplemented with 0.5% BSA and 2 mM EDTA), and applied to LS magnetic columns (Miltenyi Biotec). Positively selected CD4^+^ T cells were collected and washed prior to downstream applications. The purity of the isolated cells was evaluated by flow cytometry after staining with PE-conjugated anti-CD4 antibodies (clone L200, BD Biosciences), which are cross-reactive with Rhesus macaques. Cells were incubated with antibodies for 30 min at 4°C in the dark, washed, and analyzed on an Accuri C6 flow cytometer. Data were processed using FlowJo software (Tree Star).

### 2.4 Coculture experiment

The suppressive effect of pMregs on mitogen-induced T-cell proliferation was evaluated using pig CD4^+^ T cells. CD4^+^ T cells were isolated from the CD14-negative fraction of PBMCs by positive selection with anti-CD4 biotin and anti-biotin microbeads (Miltenyi). A total of 2 × 10^6^ CD4^+^ T cells were labeled with 1 μM CFSE (BioLegend) for 10 min at 37°C, quenched with medium containing 10% FBS, and washed three times. Labeled cells were co-cultured with pMregs at a 1:1 ratio. Both CD4^+^ T cells alone and those co-cultured with pMregs were stimulated with 2.5 μg/mL concanavalin A (ConA; Sigma-Aldrich) for 5 days and analyzed by flow cytometry.

To assess macrophage–endothelial interactions, human M1 macrophages were co-cultured with pMregs at a 1:1 ratio for 3 days without removal of pMregs. Pig endothelial MPN-3 cells were then added to the same wells (final ratio hM1: pMregs: MPN-3 = 1: 1: 1) and incubated for 6 h. Total RNA from the mixed co-culture was used to quantify human inflammatory and coagulation, and anticoagulant-related genes. hM1 cells co-cultured with HUVECs served as controls.

For cross-species assays, pMregs were co-cultured with monkey CD4^+^ T cells enriched from PBMCs, following the same protocol as for pig T cells. To test effects on human T cells, pMregs were co-cultured with Jurkat T cells, and absolute CD3^+^ T-cell numbers were determined using counting beads. T-cell inhibition was assessed by comparing cell numbers in the presence or absence of pMregs. To further investigate the underlying mechanism, we performed transwell co-culture assays in parallel to distinguish contact-dependent from soluble factor–mediated effects. The procedures and conditions are described in detail in the **Supplementary Materials**.

To evaluate induction of human regulatory T cells, pMregs were co-cultured with Jurkat T cells for 5 days. Control groups included unstimulated T cells or those stimulated with immobilized CD3 (5 μg/mL) plus soluble CD28 (5 μg/mL). After 5 days, cells were stained with CD4-PE and FOXP3-AF488, and the frequency of FOXP3^+^ regulatory T cells was determined by flow cytometry.

### 2.5 Flow cytometry

For extracellular protein expression, cells were incubated with anti-CD14 (Miltenyi), anti-CD16 (BD Biosciences), anti-CD32 (Arigo Biolaboratories), anti-CD163 (Invitrogen), PD-L1 (Proteintech), and anti-CD169 (Invitrogen) antibodies for 30 min at 4°C. For intracellular protein expression, cells were fixed and permeabilized using Cytofix/Cytoperm solution (BD Biosciences) for 20 min at 4°C, followed by staining with anti-FOXP3 (Invitrogen), anti-DHRS9 (AFG Scientific), and anti-iNOS (Santa Cruz Biotechnology) for 30 min at 4°C. After each step, samples were washed three times with DPBS. Finally, samples were resuspended in 0.4 mL DPBS and analyzed on a Cytomics FC500 MLP flow cytometer (Beckman Coulter, Fullerton, CA, USA).

### 2.6 ELISA

Concentrations of IL-10 (P1000, R&D Systems), TGF-β (DB100C, R&D Systems), and IL-1β (PLB00B, R&D Systems) in culture supernatants were quantified using commercial ELISA kits according to the manufacturer’s instructions.

### 2.7 RNA preparation and quantitative real-time PCR

Total RNA was extracted 4 h post-treatment using the easy-BLUE™ Total RNA Extraction Kit (iNtRON Biotechnology, Seoul, Korea) following the manufacturer’s protocol. RNA concentration was measured with a MaestroNano microvolume spectrophotometer (Maestrogen, Las Vegas, NV, USA). Two micrograms of RNA were reverse-transcribed into cDNA using the Hyperscript™ 2X RT Master Mix (GeneAll Biotechnology, Seoul, Korea).

qPCR was performed on a Rotor-Gene system (Qiagen) using EzAMP™ FAST One-Step RT-qPCR 2× Master Mix (SYBR; Elpis-Biotech, Daejeon, Korea). Primer sequences were as follows: IL-1β 5′- GGGATAACGAGGCTTATGTGC -3′, 5′- AGGTGGAGAGCTTTCAGTTCA-3′, IL-6 5′- GACCCAACCACAAATGCCAG -3′, 5′- GAGTTGTCATGTCCTGCAGC -3′, IL-12 5′- CATCAACCGCCAGATCAACC -3′, 5′- AGCCCATTCTCCCAGTCATC -3′, TNF-α 5′- TCTGGGCAGGTCTACTTTGG -3′, 5′- ATCCCAGGTTTCGAAGTGGT -3′, TF 5′- GGGCTGACTTCAATCCATGT -3′, 5′- GAAGGTGCCCAGAATACCAA -3′, PAR-1 5′- AGAAAGTTCCGATCCCAGCA -3′, 5′- CCCAGCAGTCCCTTTTCCTA -3′, GAPDH 5′- CACATCGCTCAGACACCATG -3′, 5′- TCCCGTTCTCAGCCATGTAG -3′, pig GAPDH 5′- ACCCAGAAGACTGTGGATGG -3′, 5′- GTCCTCAGTGTAGCCCAGGA -3′, pig DHRS9 5′- CCCAGGCTTCTAGTTTCCCA -3′, 5′- ATGACCCTTCCTTCTCCTGC -3′, pig IL-10 5′- AGAGGGGTGTCTACAAAGCC -3′, 5′- AGAGGTACAGCAGGGTTTCC -3′, pig TGF-β 5′- TGGGCCCTATGGATATGCTG -3′, 5′- AGGAAACCCATAATGCCCCA -3′, pig NOS2 5′- AAGTTAAAGCTGCCACCACG -3′, 5′- CCTGTCACCTTGGGAAGAGT -3′, pig IDO 5′- CTCCGTCCGTGAGTTTGTTC -3′, 5′- GAAGGCTCTTCGGATGCTTG -3′, pig PTGES2 5′- GGAAGGAGACAGCTTGCAAC -3′, 5′- AGAACTCGGTCACCTCCTTG -3′, pig ARG1 5′- AGGTATGAACTTGCTGGGCT -3′, 5′- CCAGTCTTCCCTCTCAGCAA -3′, pig IL-6 5′- CTAACCCCACCACAAATGCC -3′, 5′- AGTTATGTGCCCAGTGGACA -3′, pig TNF-α 5′- GCCTCTTCTCCTTCCTCCTG -3′, 5′- GCATACCCACTCTGCCATTG -3′. Results were normalized, using GAPDH as the endogenous control. Relative mRNA expression levels were normalized to GAPDH and calculated using the 2^-^ΔΔCt method.

### 2.8 Statistical analysis

All experiments were independently repeated at least three times, and data are presented as mean ± standard deviation (SD). Statistical comparisons between groups were performed using one-way analysis of variance (ANOVA) followed by a *post hoc* test (SPSS v12.0 for Windows). Differences were considered statistically significant at p < 0.05.

## 3 Results

### 3.1 Characterizations of pig regulatory macrophages

**Figure 1:**
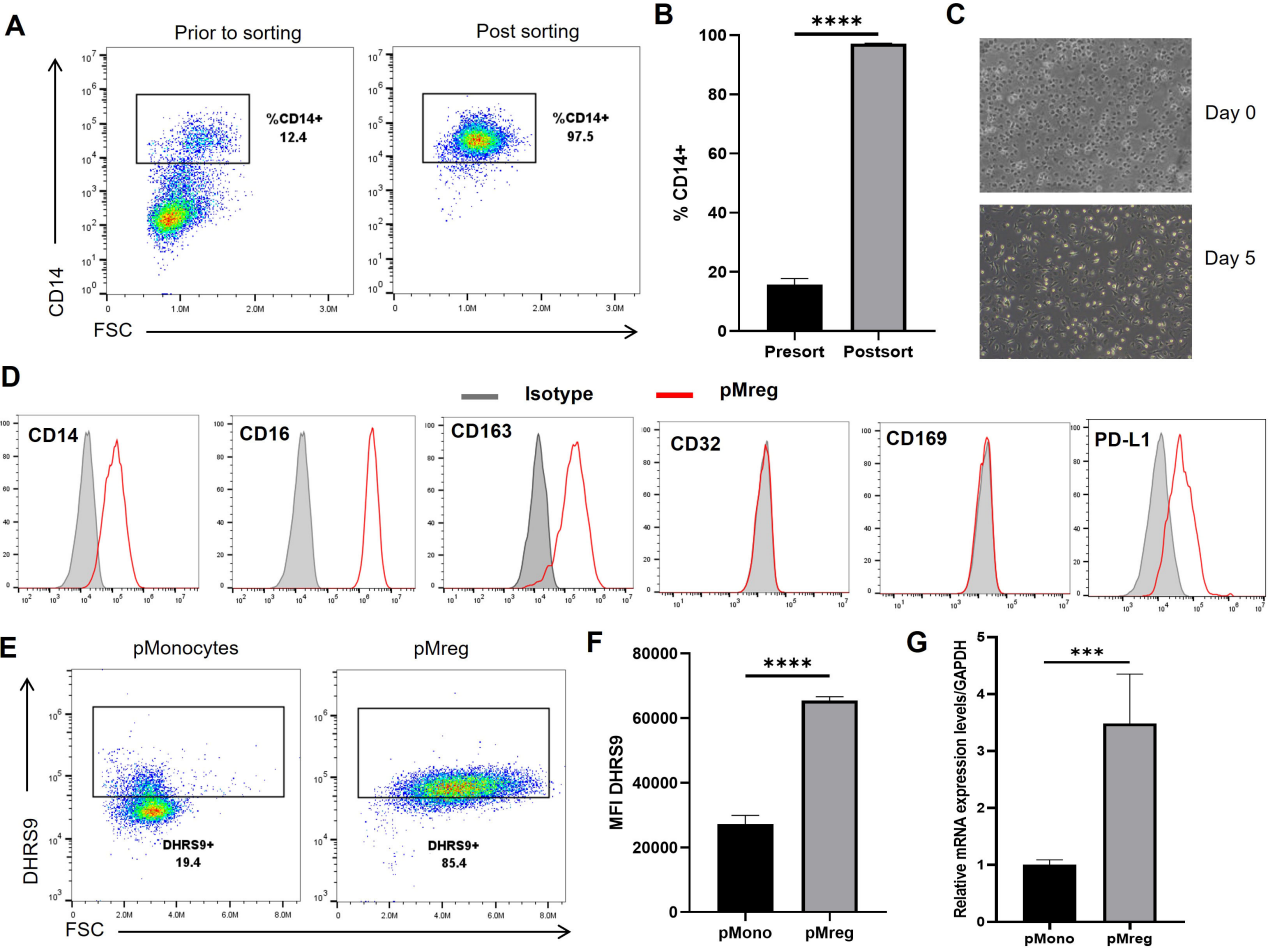
Characterization of pMregs. Pig CD14^+^ monocytes were isolated from PBMCs using anti-CD14 magnetic microbeads and cultured in RPMI-1640 supplemented with 10% pig serum, 100 U/mL penicillin, 100 μg/mL streptomycin, and 5 ng/mL M-CSF for 4 days, followed by stimulation with 25 ng/mL recombinant pig IFN-γ for 24 h. Cell morphology was assessed by inverted microscopy, and marker expression was analyzed by flow cytometry and qPCR. **(A, B)** Representative flow cytometry plots and quantification of CD14 expression before and after magnetic bead sorting. **(C)** Phase-contrast images of monocytes at Day 0 and differentiated macrophages at Day 5 (200×). **(D)** Flow cytometric histograms of surface marker expression in pMregs (red lines) compared with isotype controls (gray shading). **(E–G)** DHRS9 expression in monocytes and pMregs assessed by flow cytometry (proportion and median fluorescence intensity) and by qPCR for mRNA levels. ***p < 0.001, ****p < 0.0001.

To generate pMregs from pig monocytes, CD14^+^ cells were enriched from PBMCs by magnetic bead sorting. Flow cytometry analysis revealed that prior to enrichment, CD14^+^ cells comprised approximately 12.4% of the PBMC population, reflecting the heterogeneous mixture of lymphocytes and monocytes typically found in peripheral blood (**Figure 1A**). Following magnetic selection, the proportion of CD14^+^ cells increased to 97.5%, yielding a highly homogeneous monocyte population. Quantitative analysis confirmed a significant improvement in purity after sorting (**Figure 1B**). Morphological changes during differentiation were observed in culture. On Day 0, freshly isolated monocytes displayed a small, round, and non-adherent morphology. After 5 days of culture with M-CSF, followed by stimulation with IFN-γ, the cells became adherent, spread, and elongated—features typical of macrophage differentiation (**Figure 1C**), consistent with previous descriptions (22).

We next examined the marker expression profile of pMregs. Flow cytometry revealed strong expression of anti-inflammatory markers (CD163, PD-L1) and phagocytosis-associated markers (CD14, CD16), while CD32 and CD169 were absent (**Figure 1D**). A similar expression pattern has been reported for human Mregs (15, 20), suggesting a conserved marker signature across species. In addition to surface markers, we investigated the intracellular marker DHRS9, previously identified in human Mregs differentiated with M-CSF and IFN-γ (16). DHRS9 expression was assessed in pig monocytes (pMono) and pMregs at both mRNA and protein levels. Flow cytometry demonstrated a marked increase in DHRS9 expression in pMregs, with 85.4% of cells staining positive (**Figure 1E**), accompanied by a significant rise in median fluorescence intensity (**Figure 1F**). qPCR analysis further confirmed these results, showing a 3.4-fold upregulation of DHRS9 mRNA in pMregs compared with pMono (**Figure 1G**).

### 3.2 Pig Mregs upregulated anti-inflammatory cytokine IL-10, TGF-β

**Figure 2:**
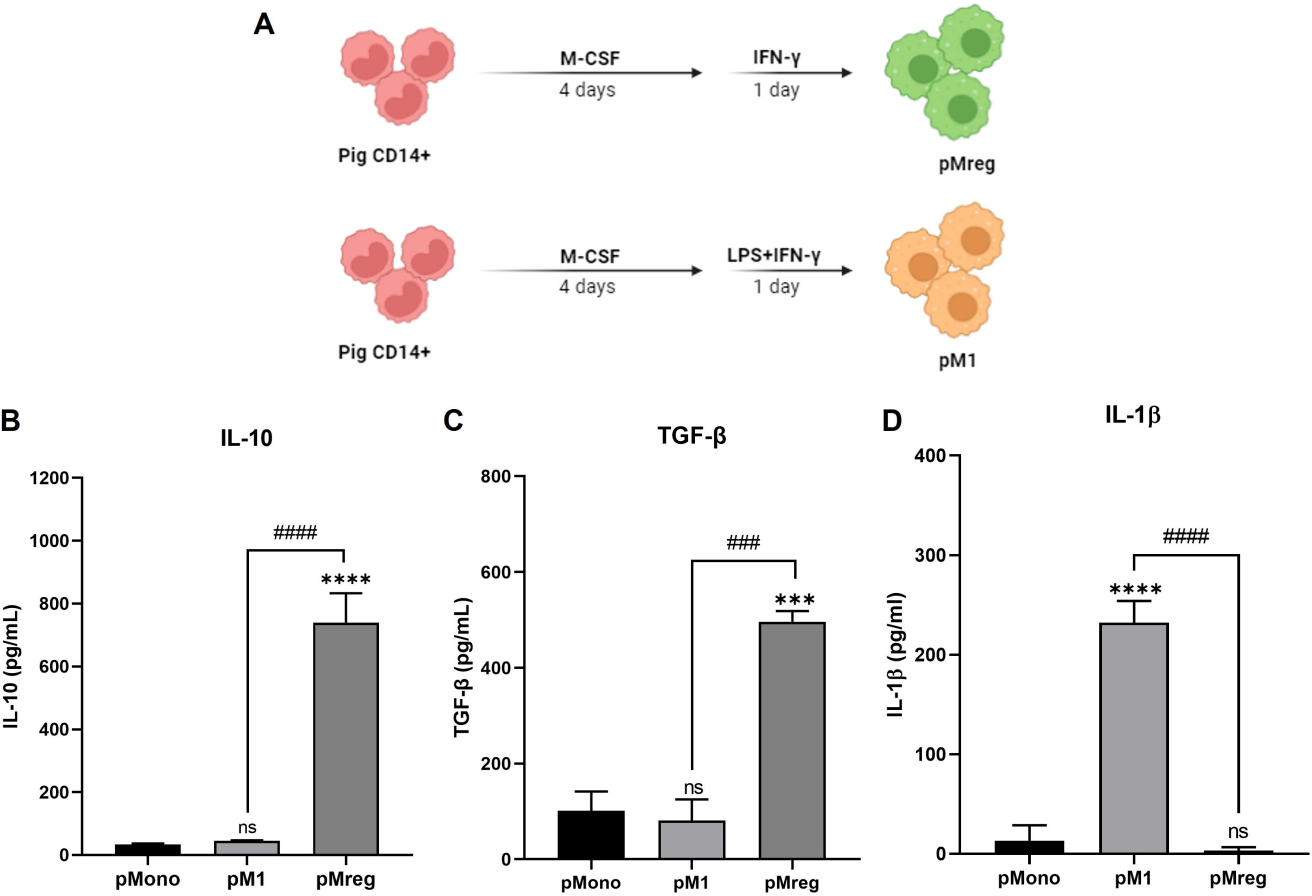
pMregs upregulate anti-inflammatory cytokines IL-10 and TGF-β. After differentiation, cells were cultured for 2 days in RPMI-1640 supplemented with 10% pig serum, 100 U/mL penicillin, and 100 μg/mL streptomycin. Supernatants were collected and analyzed for IL-10, TGF-β, and IL-1β secretion by ELISA. **(A)** Schematic representation of pMreg and pM1 generation. **(B)** Quantification of IL-10 secretion. **(C)** Quantification of TGF-β secretion. **(D)** Quantification of IL-1β secretion. ***p < 0.001, ****p < 0.0001 vs. monocytes. ^###^p < 0.001, ^####^p < 0.0001.

The immunoregulatory capacity of macrophages is closely associated with their secretion of anti-inflammatory cytokines (23). To assess this, we measured the secretion of IL-10 and TGF-β, as well as the pro-inflammatory cytokine IL-1β, by pMonos, pM1 macrophages, and pMregs using ELISA (**Figure 2A**). pMregs secreted significantly higher levels of the anti-inflammatory cytokines IL-10 and TGF-β compared with both undifferentiated pMonos and pM1 macrophages (**Figures 2B, C**). In contrast, pMregs produced only low levels of IL-1β, whereas pM1 macrophages secreted abundant IL-1β, consistent with their pro-inflammatory phenotype (**Figure 2D**).

To further assess inflammatory signaling, IL-6 and TNF-α mRNA levels were measured by qRT-PCR. pM1 macrophages showed strong induction of both cytokines, whereas Mregs exhibited markedly lower IL-6 expression and minimal TNF-α upregulation (**Supplementary Figure S4**), consistent with their anti-inflammatory phenotypes.

### 3.3 Pig Mregs inhibit pig CD4^+^ T cell proliferation and induce the differentiation of pig FOXP3^+^ Tregs

**Figure 3:**
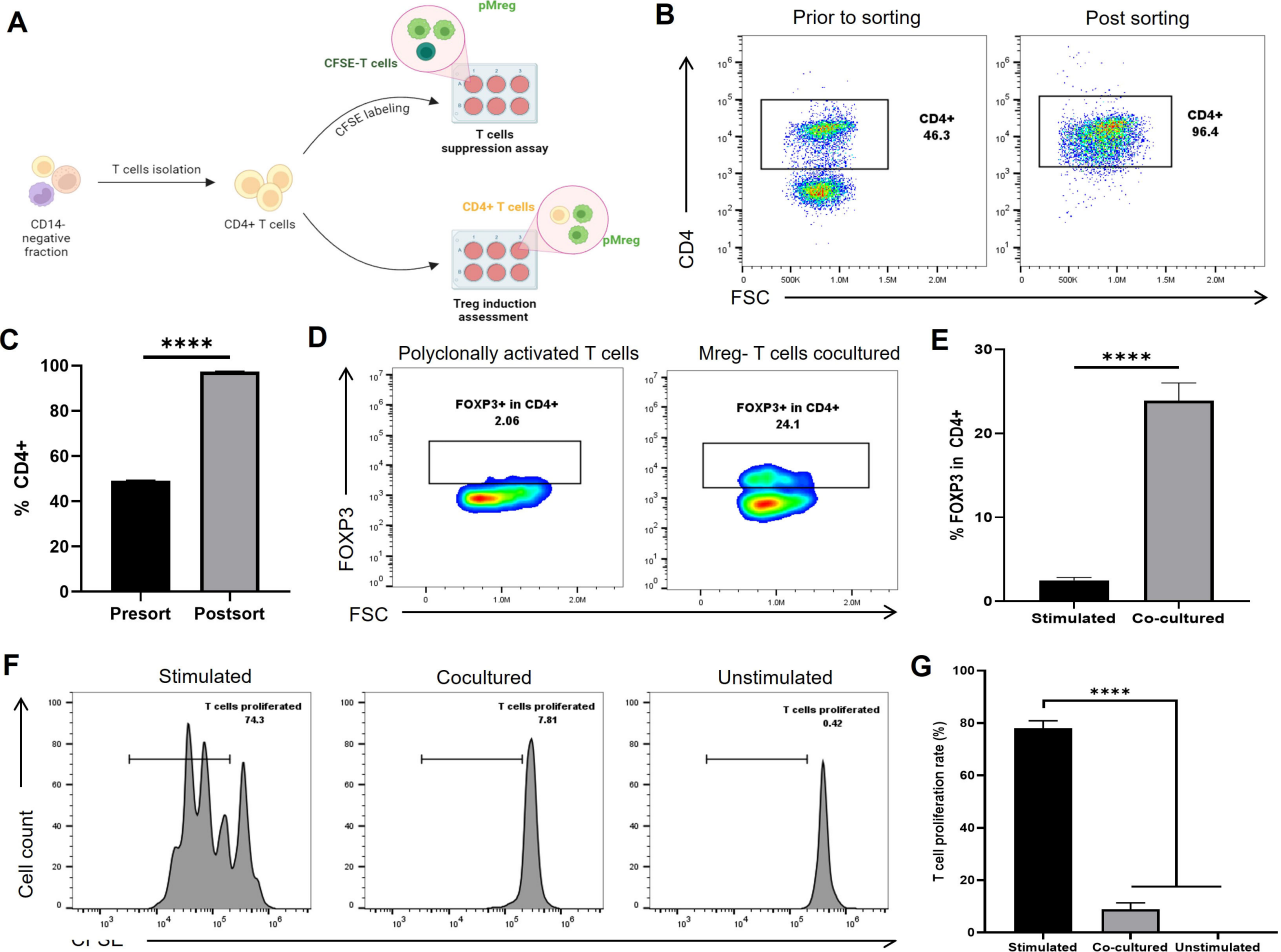
pMregs inhibit pig CD4^+^ T-cell proliferation and induce differentiation of FOXP3^+^ Tregs. pMregs were co-cultured with CFSE-labeled pig CD4^+^ T cells for 5 days. T cells were stimulated with ConA (2.5 µg/mL), harvested, and analyzed by flow cytometry. **(A)** Experimental schematic illustrating the assessment of pMreg-mediated inhibition of T-cell proliferation and induction of Tregs. **(B, C)** Representative flow cytometry plots showing CD4^+^ T-cell populations before and after CD4 enrichment. **(D, E)** Pig CD4^+^ T cells co-cultured with pMregs exhibited increased FOXP3^+^ T-cell enrichment compared with ConA-stimulated CD4^+^ T cells alone. **(F, G)** Proliferation of CFSE-labeled CD4^+^ responder T cells stimulated with ConA was significantly inhibited in the presence of pMregs (mean ± SD). ****p < 0.0001.

The immunomodulatory functions of pMregs were evaluated in a co-culture model with pig CD4^+^ T cells (**Figure 3A**). CD4^+^ T cells were enriched from the CD14^-^ fraction with >95% purity (**Figures 3B, C**). Cells were either labeled with CFSE to assess proliferation or directly co-cultured with pMregs for 5 days to evaluate regulatory T-cell (Treg) induction. Co-culture with pMregs resulted in a marked enrichment of FOXP3^+^ T cells, a hallmark of Tregs (24), compared with both unstimulated controls and cells stimulated with anti-CD3/CD28 in the absence of pMregs (**Figures 3D, E**). To assess the ability of pMregs to suppress T-cell proliferation, CD4^+^ T cells were stimulated with the mitogen ConA (25). As expected, ConA stimulation alone induced robust T-cell proliferation (**Figure 3F**). In contrast, co-culture with pMregs at a 1:1 ratio strongly inhibited proliferation, with only ~7% of T cells dividing despite ConA stimulation (**Figure 3G**). These findings demonstrate the potent immunosuppressive capacity of pMregs in regulating T-cell responses.

### 3.4 pMregs suppress pro-inflammatory and coagulation-related gene expression in human macrophage–pig endothelial cell co-cultures

In xenotransplantation, recognition of pig antigens by recipient immune cells is an early trigger of inflammatory and pro-coagulant pathways. To model this interaction, we established three co-culture conditions with the pig endothelial cell line MPN3: (1) hM1 macrophages directly co-cultured with MPN3, (2) hM1 pre-exposed to pM1 for 3 days before co-culture with MPN3, and (3) hM1 pre-exposed to pMregs for 3 days before co-culture with MPN3. Co-culture of hM1 with human endothelial cells (HUVECs) served as the control. mRNA expression of pro-inflammatory cytokines (IL-1β, IL-6, IL-12, TNF-α) (21) and coagulation-related genes (PAR-1, TF) (26, 27) was subsequently assessed.

**Figure 4:**
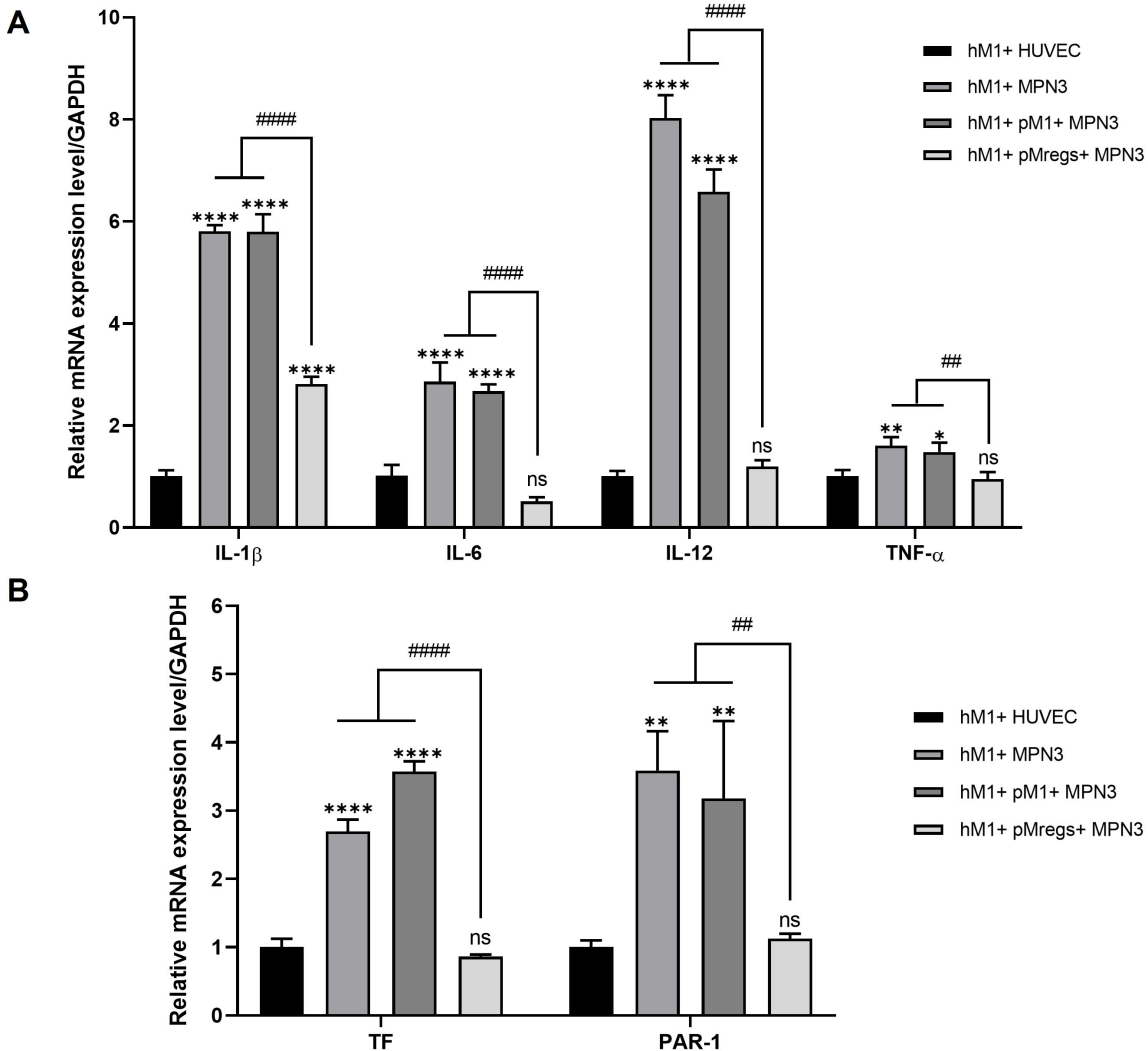
pMregs suppress mRNA expression of pro-inflammatory and coagulation-related genes in hM1–MPN3 co-cultures. pMregs and pM1 were generated as described in the Methods. hM1 were generated from THP-1 cells by treatment with 10 ng/mL PMA for 2 days, followed by 100 ng/mL LPS and 20 ng/mL IFN-γ. Thereafter, hM1 were co-cultured with either pM1 or pMregs for 3 days before incubation with MPN3 cells for 6 h; hM1 co-cultured with HUVECs served as the control. After incubation, cells were harvested, and mRNA expression of cytokines **(A)** and coagulation-related genes **(B)** was analyzed by qRT-PCR. **p < 0.01, ****p < 0.001 vs. hM1 + HUVEC; ^##^p < 0.01, ^####^p < 0.0001 vs. hM1 + pMregs + MPN3.

As shown in **Figure 4A**, hM1 co-cultured with MPN3 alone or pre-exposed to pM1 exhibited marked upregulation of IL-1β, IL-6, IL-12, and TNF-α compared with the hM1 + HUVEC control. In contrast, pre-exposure of hM1 to pMregs prior to MPN3 co-culture significantly suppressed the expression of these cytokines. A similar pattern was observed for coagulation-related genes (**Figure 4B**). hM1 co-cultured with pM1 and MPN3 showed elevated TF and PAR-1 expression relative to the control, whereas pre-exposure to pMregs nearly abolished their expression, reducing both to levels comparable to the control group. Collectively, these findings indicate that pMregs effectively attenuate both inflammatory and pro-thrombotic responses in hM1 during pig–human cellular interactions, supporting their potential role in mitigating graft rejection in xenotransplantation.

### 3.5 pMregs inhibit monkey and human T-cell proliferation and promote FOXP3^+^ Jurkat T-cell differentiation

**Figure 5:**
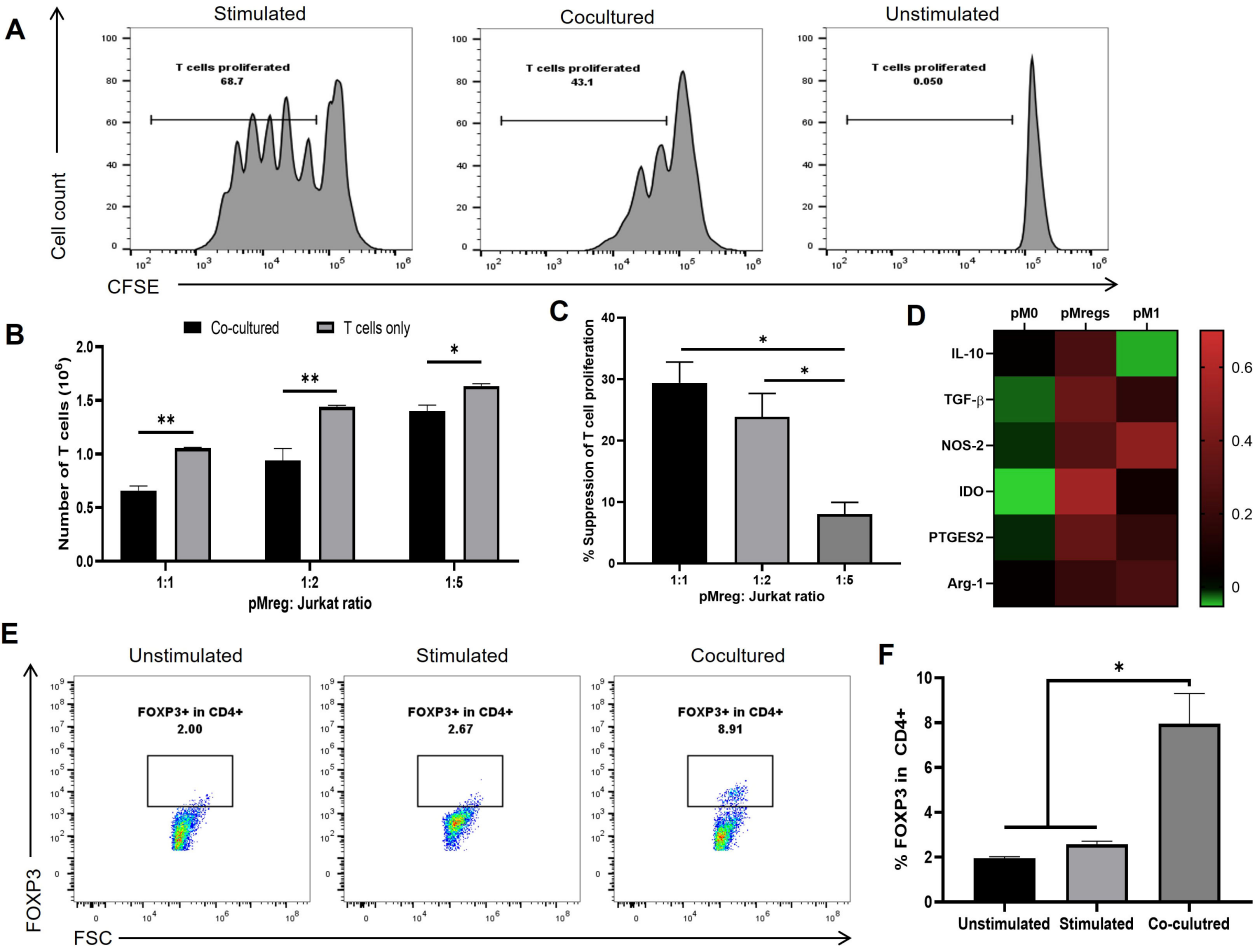
pMregs inhibit proliferation of monkey and human T cells and promote FOXP3^+^ Jurkat T-cell differentiation. pMregs were co-cultured with CFSE-labeled CD4^+^ T cells from monkey PBMCs or with human Jurkat T cells under different conditions. For monkey T cells, CD4^+^ T cells were stimulated with ConA (2.5 µg/mL) in the presence or absence of pMregs for 5 days, and proliferation was assessed by flow cytometry. For human T cells, Jurkat cells were co-cultured with pMregs at varying ratios. After 3 days, absolute cell numbers were determined using counting beads, and after 5 days, FOXP3 expression was analyzed by flow cytometry to evaluate regulatory T-cell induction. **(A)** CFSE profiles showing suppression of ConA-induced proliferation of monkey CD4^+^ T cells by pMregs. **(B)** Absolute numbers of Jurkat T cells after 3 days of co-culture with or without pMregs at different ratios. **(C)** Quantification of the suppressive effect of pMregs on Jurkat T-cell proliferation at increasing T cell:pMreg ratios. **(D)** Expression of canonical Mreg effector pathways in porcine macrophage subsets. Heatmap showing median-centered log2 expression values of IL-10, TGF-*β*, NOS-2, IDO, PTGES2, and Arg1 in pM1, pMregs, and pM0. Gene expression was quantified by qRT-PCR using species-specific primers. **(E, F)** Flow cytometry plots and quantification of FOXP3^+^ Jurkat T cells after 5 days of co-culture with pMregs. *p < 0.05 **p < 0.01. pM0: unstimulated macrophages; pMregs: M-CSF/IFN-γ-induced regulatory macrophages: pM1: LPS+IFN-γ-stimulated pro-inflammatory macrophages.

T-cell–mediated responses represent a major barrier in xenotransplantation. To assess the immunosuppressive capacity of pMregs, we evaluated their effects on primary monkey CD4^+^ T cells and human Jurkat T cells. In monkey CD4^+^ T cells isolated from PBMCs, ConA stimulation induced robust proliferation, whereas co-culture with pMregs resulted in a moderate but consistent reduction in ConA-induced proliferation of monkey CD4+ T cells, as shown by the decreased CFSE dilution compared with control (**Figure 5A**). Similarly, pMregs induced moderate suppression of Jurkat T-cell proliferation at lower T cell:pMreg ratios, with the inhibitory effect decreasing at higher Jurkat cell densities, indicating a dose-dependent response (**Figures 5B, C**). Regulatory T cells (Tregs) play a key role in maintaining immune tolerance (28). To evaluate the capacity of pMregs to induce Tregs, Jurkat CD4^+^ T cells were co-cultured with pMregs for 5 days. FOXP3 expression, analyzed by flow cytometry, remained low in both unstimulated controls (2%) and anti-CD3/CD28–stimulated T cells (2.67%). In contrast, co-culture with pMregs significantly increased the proportion of FOXP3^+^ CD4^+^ T cells (8.91%) (**Figures 5E, F**). These findings indicate that pMregs not only suppress T-cell proliferation across species but also promote the differentiation of regulatory T cells, thereby reinforcing their immunoregulatory function.

To further characterize canonical Mreg effector pathways, we analyzed the expression of IL-10, TGF-β, NOS-2, IDO-1, PTGES2, and Arg-1 in pM0, pMregs, and pM1 using species-specific qRT-PCR. As shown in **Figure 5D**, pMregs expressed detectable levels of all assessed genes. IL-10 and IDO1 were elevated in pMregs compared with pM0, while NOS-2 and PTGES2 showed modest increases. In contrast, Arg-1 expression remained low across all macrophage subsets. These finding indicate that pMregs exhibit moderate activation of several canonical Mreg effector pathways compared to pM1 profiles.

**Figure 6:**
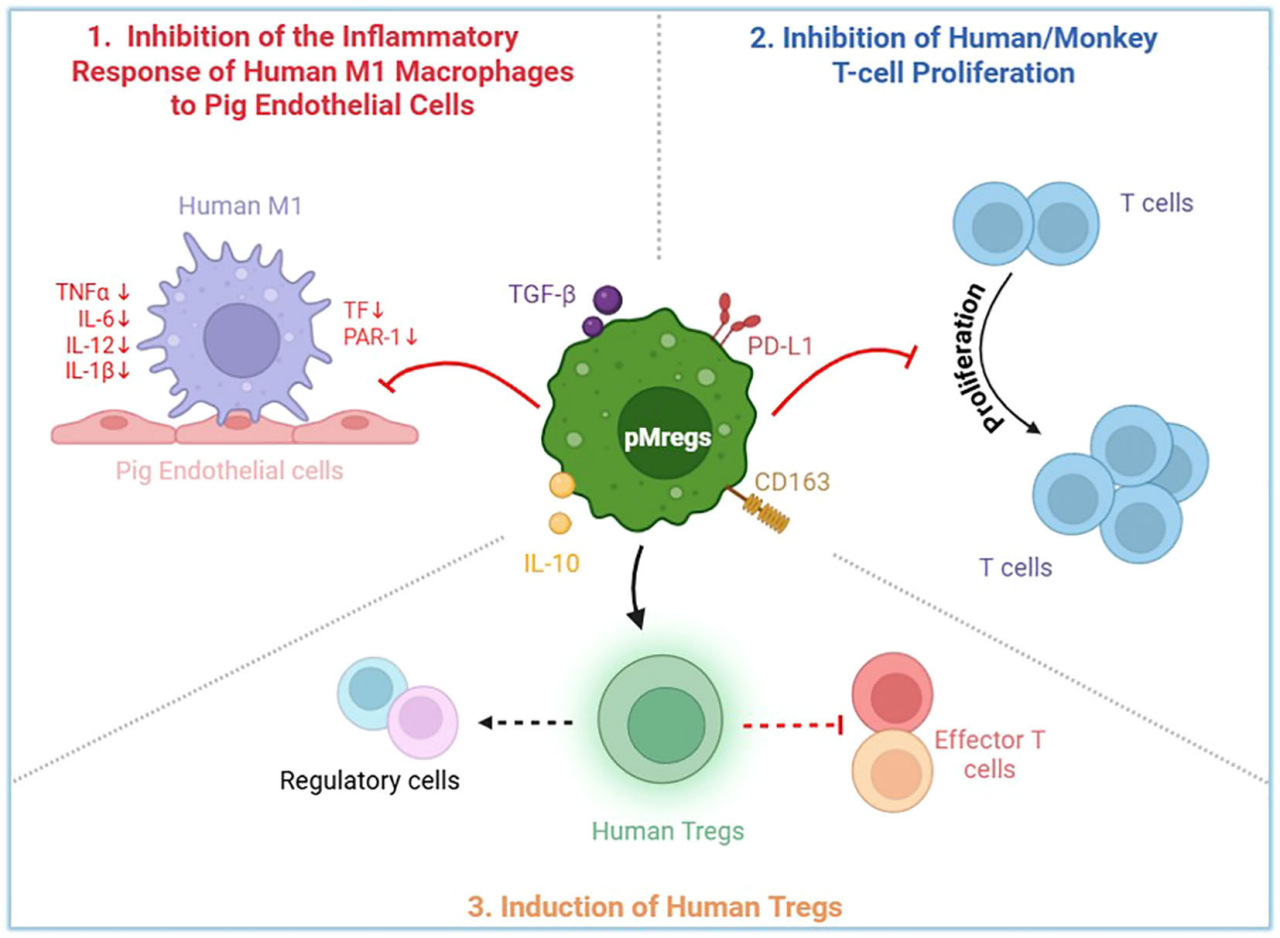
Schematic representation of the immunoregulatory functions of pMregs in xenotransplantation. Based on *in vitro* results, pMregs exert multiple immunomodulatory effects across innate and adaptive immune responses. (1) pMregs suppress pro-inflammatory responses of human M1 macrophages stimulated by pig endothelial cells, leading to downregulation of cytokines (TNF-α, IL-6, IL-12, IL-1β) and coagulation-associated molecules (TF, PAR-1). (2) pMregs inhibit the proliferation of human and monkey T cells. (3) pMregs promote the induction of FOXP3^+^ human regulatory T cells. Collectively, these mechanisms highlight the potential of pMregs to reduce xenogeneic immune activation and promote tolerance in xenotransplantation.

Taken together, these findings demonstrate that pMregs exert multifaceted immunoregulatory functions. As summarized in **Figure 6**, pMregs (1) suppress pro-inflammatory and pro-coagulant responses of human M1 macrophages to pig endothelial cells, (2) inhibit the proliferation of human and monkey T cells, and (3) promote the induction of FOXP3^+^ regulatory T cells, collectively supporting their potential to attenuate xenogeneic immune activation.

## 4 Discussion

In this study, we investigated the immunoregulatory potential of pMregs in the context of xenotransplantation. Our findings demonstrate that pMregs exhibit broad immunomodulatory activity, effectively suppressing the proliferation of T cells derived from pigs, nonhuman primates, and humans. In addition, pMregs attenuated pro-inflammatory M1 macrophage activity upon interaction with pig endothelial cells. Together, these *in vitro* results suggest that pMregs can mitigate xenogeneic immune activation, thereby pointing to a potential strategy for reducing rejection risk and favoring a tolerogenic environment.

Phenotypically, pMregs generated from PBMC-derived CD14^+^ monocytes under M-CSF and IFN-γ stimulation displayed elongated morphology and exhibited features comparable to regulatory macrophages previously reported in humans and mice, sharing a typical regulatory macrophage signature that included the expression of CD14^+^, CD16^+^, CD163^+^, PD-L1^+^, and DHRS9^+^ (20, 29, 30). This overlap indicates that pMregs belong to the broader category of regulatory macrophages. At the same time, pMregs consistently lacked CD32 and CD169 expression, a distinct pattern that represents a species-specific difference and sets them apart from their human counterparts. Several molecules within this profile underscore their immunosuppressive potential: PD-L1 functions as a crucial checkpoint ligand that inhibits T-cell activation (31), CD163 is associated with anti-inflammatory macrophage subsets (32), and DHRS9 has been identified as a defining marker of regulatory macrophages (16). These similarities strengthen the translational relevance of pMregs across species.

Functionally, pMregs secreted IL-10 and TGF-β, key cytokines known to suppress inflammation and support graft survival (33). TGF-β contributes to T-cell suppression and maintenance of immune homeostasis (34), while IL-10 inhibits pro-inflammatory T-cell responses and promotes Treg differentiation (35). In line with this cytokine profile, pMregs facilitated the induction of FOXP3+ Tregs, a population widely recognized as essential for durable graft tolerance (36). Furthermore, pMregs reduced inflammatory cytokines such as IL-1β and IL-6 in human M1 macrophages (37, 38), supporting their anti-inflammatory role. Because Jurkat cells differ fundamentally from primary human T cells in their activation thresholds and transcriptional programs, the Jurkat-based findings in this study should be interpreted as preliminary indicators of regulatory signaling rather than definitive evidence of human T-cell suppression or Treg induction.

While these findings are compatible with PD-L1-associated and cytokine-driven regulatory mechanisms previously reported in human Mregs (39), direct pathway validation was not feasible in this study because cross-reactive pig-human blocking antibodies for PD-1/PD-L1, IL-10, or TGF-β are not commercially available. To further examine the mode of suppression in the absence of such reagents, we performed transwell co-culture assay (**Supplementary Figure S2**). pMregs retained substantial suppressive activity under non-contact conditions, indicating a role for soluble mediators. At the same time, the reduction magnitude of suppression compared with direct co-culture suggests that contact-dependent mechanisms may also contribute, consistent with the observed surface expression of PD-L1 and other regulatory markers. Together, these data support a model in which pMregs-mediated immunomodulation involves both paracrine and contact-associated components, although definitive pathway dissection will require future studies incorporating species-matched blocking tools.

Importantly, our *in vitro* results demonstrated that pMregs possess both cross-species immunoregulatory activity – such as inhibiting the proliferation of pig, primate, and human T cells and promoting FOXP3+ Treg induction – and a capacity to reduce the transcriptional levels of TF and PAR-1 in human M1 macrophages during pig-human co-culture. While these combined features have not been previously characterized in detail for pig or human Mregs, our finding support defining the pMreg phenotype as “CD14^+^CD16^+^CD163^+^PD-L1^+^DHRS9^+^ expression with consistent CD32^-^ and CD169^-^ status, cross-species T-cell suppression, and regulation of coagulation pathways”. These features highlight the potential relevance of pMregs as donor-derived immunoregulatory cells that may complement genetically engineered pig grafts in xenotransplantation. Given the preliminary nature of the coagulation findings, we acknowledge that protein-level and functional assays – such as TF surface expression, Factors Xa activity, and thrombin generation – will be required to confirm their biological relevance.

Given the limitations of genetic engineering alone, combining CRISPR-based organ modifications with pMreg infusion could provide a synergistic strategy to overcome barriers in xenotransplantation (40). Genome editing reduces baseline immunogenicity, whereas pMregs actively establish and sustain tolerance, potentially reducing the requirement for long-term systemic immunosuppression.

Despite these promising findings, several important issues remain to be addressed before clinical application. First, although co-culture experiments suggested that human macrophages did not exhibit overt inflammatory responses to pMregs (**Figure 4**), it remains to be demonstrated *in vivo* whether the recipient’s innate immune response against infused pMregs can indeed be effectively suppressed. Second, the *in vivo* survival, stability, and homing capacity of pMregs to graft sites are still unclear, leaving questions regarding their long-term regulatory function. Third, it is necessary to evaluate how pMregs interact with conventional immunosuppressive agents, as combination regimens will likely be required in practical xenotransplantation protocols. In addition, because this study relied on THP-1-derived macrophages and Jurkat T cells for standardized cross-species assays, confirming key findings using primary human monocyte-derived macrophages and primary CD4+ T cells will be essential in future studies to strengthen translational relevance. Finally, large-scale production of pMregs that complies with GMP standards will be essential, requiring standardized cytokine sources, culture conditions, and quality-control procedures.

In conclusion, our *in vitro* study demonstrates that pMregs possess a dual capacity to suppress xenogeneic T-cell responses across species and to regulate coagulation-related pathways, thereby suggesting a potential to address two major barriers unique to xenotransplantation. Unlike human primary Mregs, which have been primarily characterized in allotransplantation contexts, pMregs extend the current concept of regulatory macrophages by incorporating these functional hallmarks into their definition. Thus, pMregs should not only be defined by their phenotypic markers (CD14^+^CD16^+^CD163^+^PD-L1^+^DHRS9^+^CD32^-^CD169^-^) but also by their ability to inhibit cross-species T-cell proliferation and to downregulate TF and PAR-1 expression. These distinctive features highlight pMregs as a novel donor-derived immunoregulatory candidate that could complement genetically engineered grafts and potentially reduce the need for long-term immunosuppression in xenotransplantation.

## Data Availability

The original contributions presented in the study are included in the article/**Supplementary Material**. Further inquiries can be directed to the corresponding author.
