# Supplementary material for: Pig regulatory macrophages as a donor-derived immune modulators in xenotransplantation
Source: Front Immunol. 2025 Dec 5;16:1718937. doi: 10.3389/fimmu.2025.1718937 (PMC12714666; doi:10.3389/fimmu.2025.1718937)
Supplement: Supplementary file 1 [file DataSheet1.docx]

*
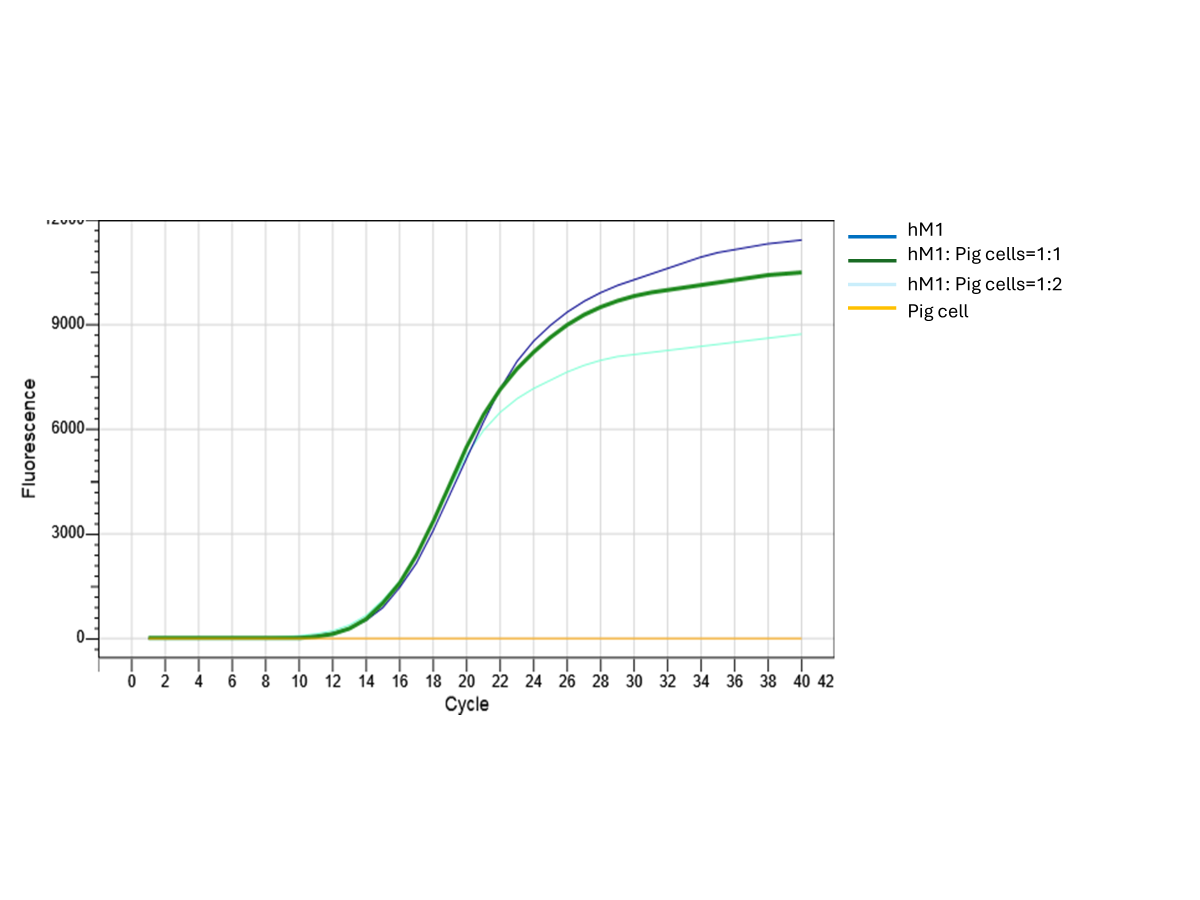
*

**Figure S1. Species-specific validation of human qPCR assays**.

Amplification plots generated using human qPCR primer (GAPDH) tested on cDNA from human M1 macrophages, pig endothelial cells (MPN-3), pig Mregs, and mixed human-pig cDNA samples (1:1 and 1:2 ratios). The curves represent amplification profiles for each primer set across the different cDNA sources.

*
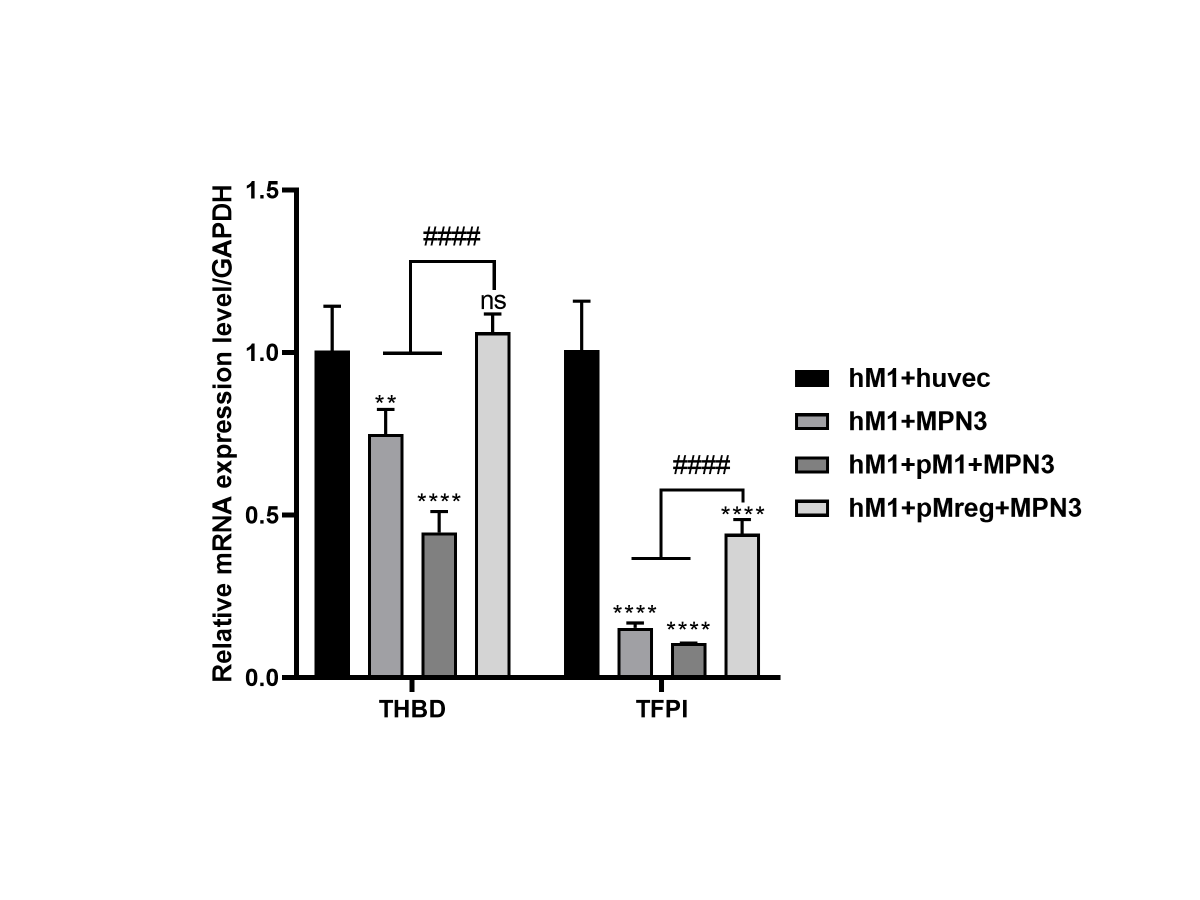
*

**Figure S2. pMregs enhance expression of coagulation-inhibitory factors in response to human M1 macrophages.** All experiments were conducted three times (n = 3). Data were presented as Mean ± SD. **p < 0.01, ****p < 0.001 vs. hM1 + HUVEC; ####p < 0.0001 vs. hM1 + pMregs + MPN3.

***
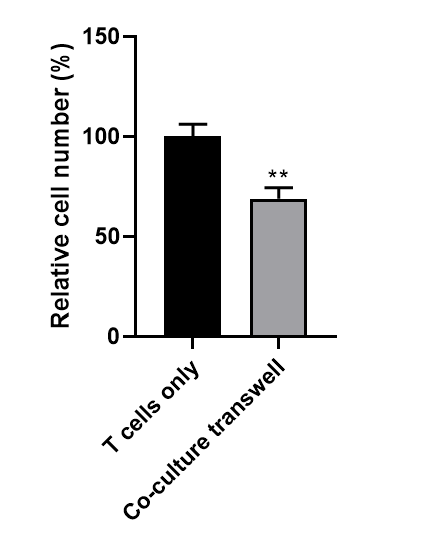
***

**Fig. S3. pMregs suppress human T cell line proliferation in transwell co-culture**

After differentiation, pMregs were co-cultured with human Jurkat T cells in a transwell system at a ratio of 1:1 for 3 days. Subsequently, T cells were collected, and their numbers were quantified using counting beads by flow cytometry. Jurkat T cells cultured alone served as the control group. Experiment were conducted three times (n = 3). Data were presented as Mean ± SD. **p < 0.01


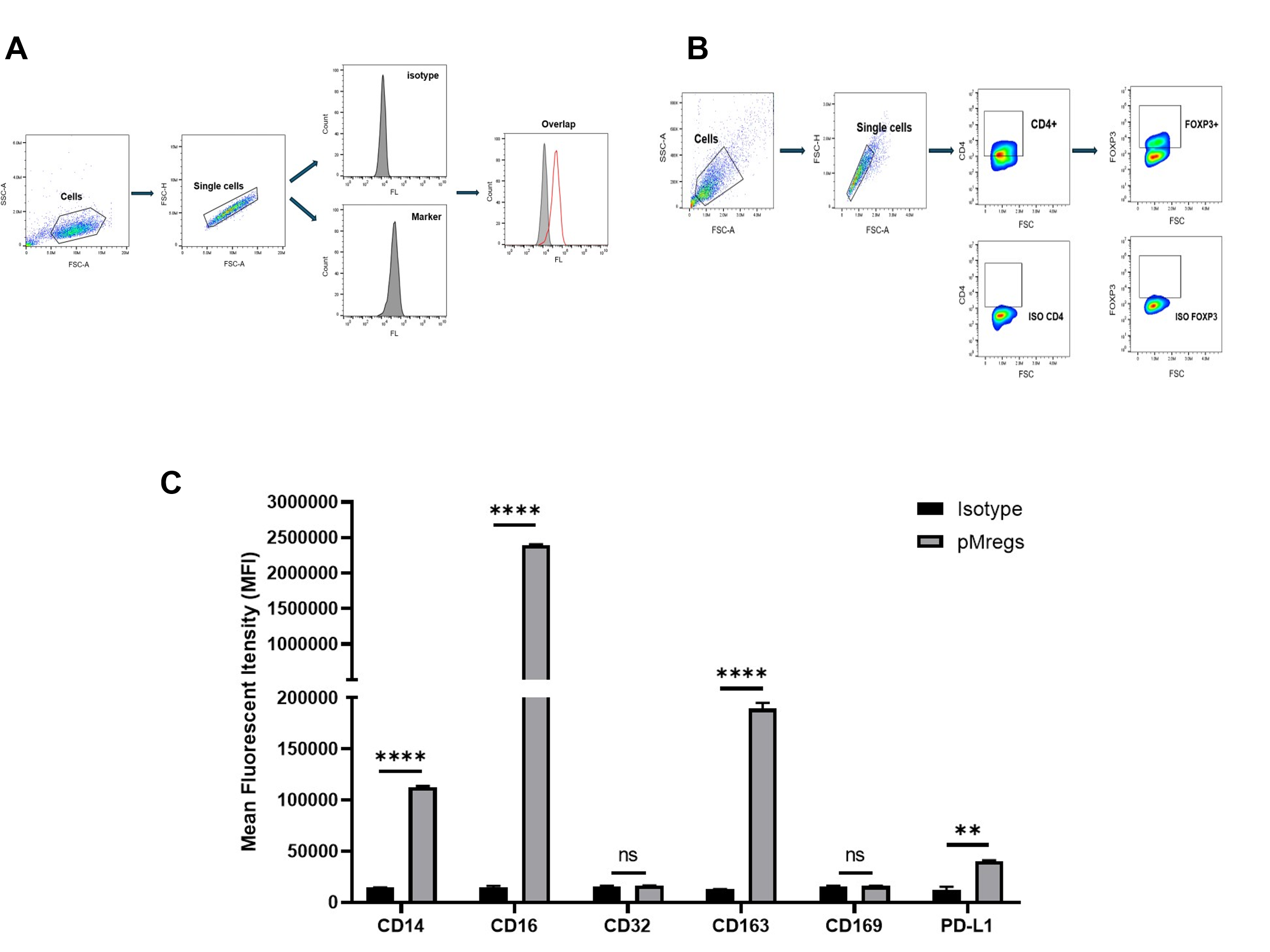
**Fig.S4. (A)** Gating strategy for the analysis of human regulatory macrophages (M regs) by flow cytometry. **(B)** Gating strategy for the analysis of FOXP3 expression. **(C)** Mean fluorescent intensity of marker expression on pMregs. All experiments were conducted three times (n = 3). Data were presented as Mean ± SD **p < 0.01, ****p < 0.001.

*
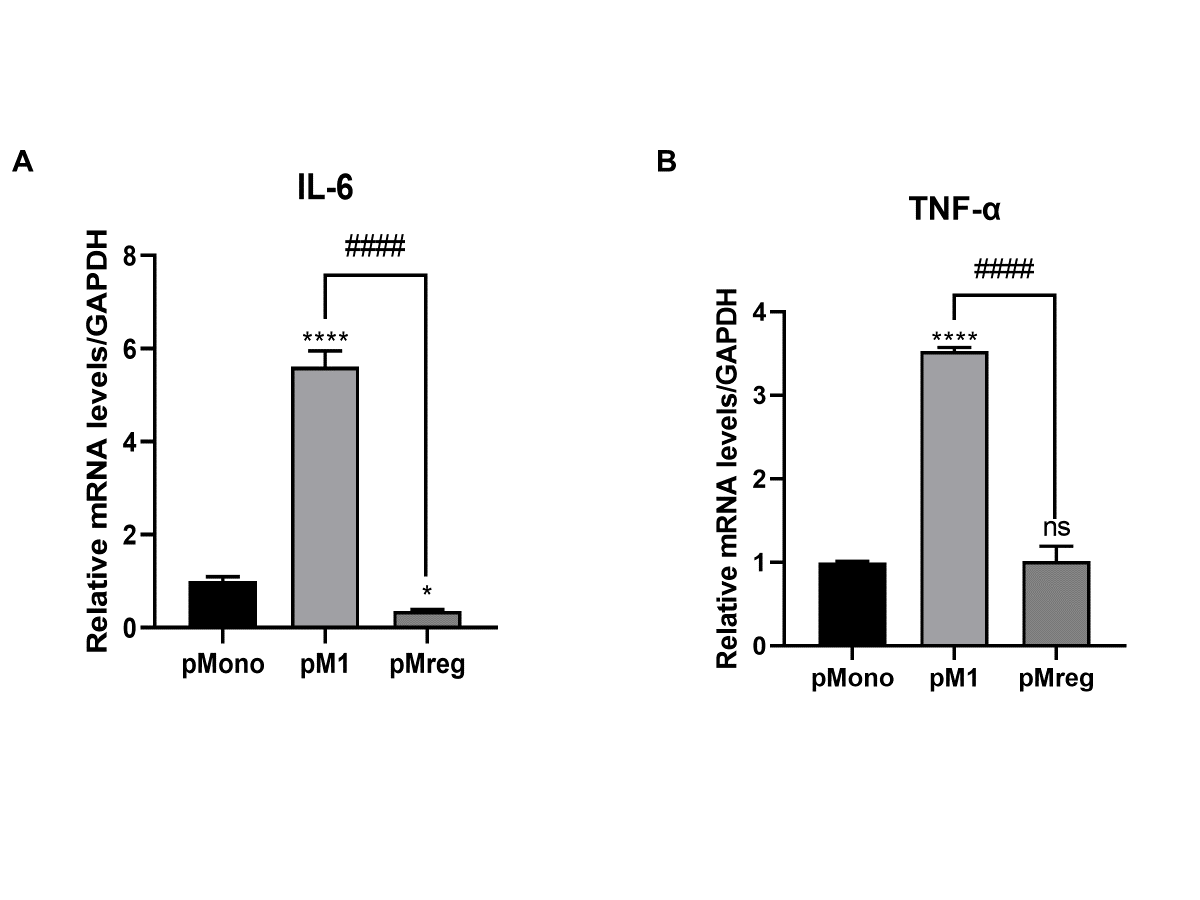
*

**Figure.S5**. **pMregs downregulate of inflammatory cytokine**. After differentiation, RNA were extracted and the relative mRNA levels of inflammatory cytokine-related gene IL-6 **(A)** and TNF-α **(B)** were assessed by qRT-PCR. All experiments were conducted three times (n = 3). Data were presented as Mean ± SD ****p < 0.001 vs.pMono; ####p < 0.0001


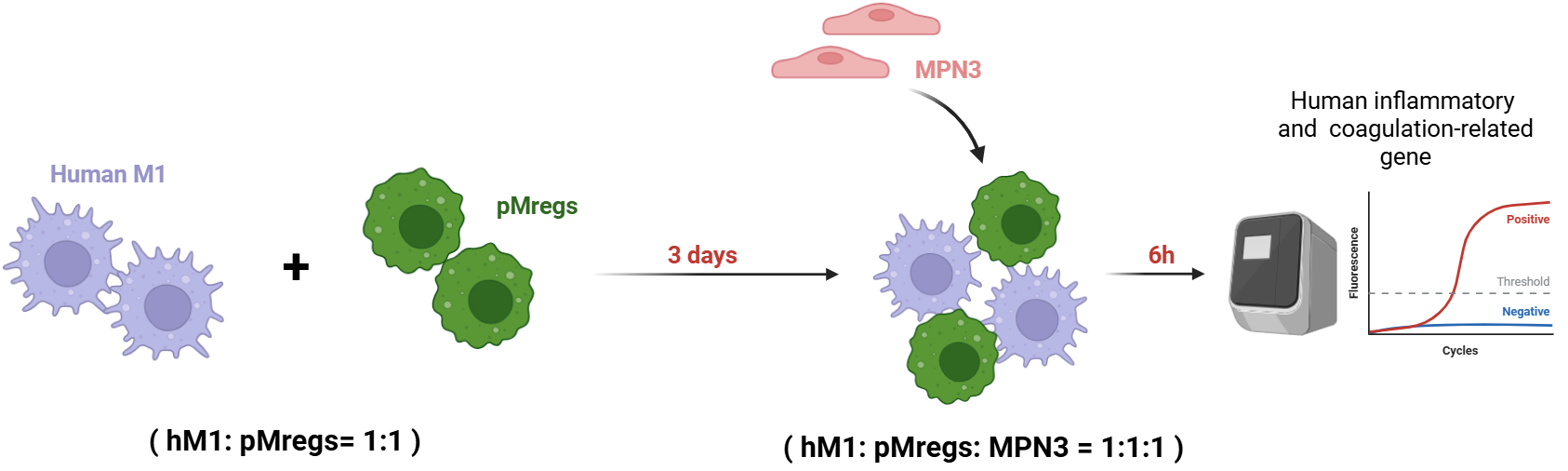


**Fig. S6. Schematic of the human M1–pMreg–MPN3 co-culture experiment for assessment of human inflammatory and coagulation-related gene expression.** Human M1 macrophages were co-cultured with pMregs at a 1:1 ratio for 3days without removal of pMregs. Thereafter, pig endothelial MPN-3 cells were added to the same wells (final ratio hM1: pMregs: MPN-3 = 1: 1: 1) and incubated for 6 h. Human inflammatory and coagulation-related gene expression was subsequently assessed by qRT-PCR from total RNA extracted from the co-culture.

**Table S1**. **List of Antibodies use in this study**

| Antibodies | Clone | Supplier | Catalog |
| --- | --- | --- | --- |
| CD14 | TÜK4 | Miltenyi Biotec | 130-113-145 |
| CD16 | FcG7 | BD Biosciences | 551395 |
| CD32 | AT10 | Arigobio | ARG22890 |
| CD163 | 2A10/11 | Invitrogen | MA5-28293 |
| CD169 | 3B11/11 | Invitrogen | MA5-28295 |
| DHRS9 | A19441 | AFG Scientific | A19441 |
| FOXP3 | FJK-16s | eBioscience™ | 14-5773-82 |
| PD-L1 | 2B11D11 | Proteintech | 66248-1-Ig |
